# Suppressing Hepatic UGT1A1 Increases Plasma Bilirubin, Lowers Plasma Urobilin, Reorganizes Kinase Signaling Pathways and Lipid Species and Improves Fatty Liver Disease

**DOI:** 10.3390/biom13020252

**Published:** 2023-01-29

**Authors:** Evelyn A. Bates, Zachary A. Kipp, Genesee J. Martinez, Olufunto O. Badmus, Mangala M. Soundarapandian, Donald Foster, Mei Xu, Justin F. Creeden, Jennifer R. Greer, Andrew J. Morris, David E. Stec, Terry D. Hinds

**Affiliations:** 1 Department of Pharmacology and Nutritional Sciences, University of Kentucky, Lexington, KY 40508, USA; 2 Department of Physiology & Biophysics, Cardiorenal and Metabolic Diseases Research Center, University of Mississippi Medical Center, Jackson, MS 39216, USA; 3 Alnylam Pharmaceuticals, Cambridge, MA 02142, USA; 4 Department of Neurosciences, University of Toledo College of Medicine and Life Sciences, Toledo, OH 43614, USA; 5 Department of Pharmacology and Toxicology, University of Arkansas for Medical Sciences, Little Rock, AR 72205, USA; 6 Barnstable Brown Diabetes Center, University of Kentucky, Lexington, KY 40508, USA; 7 Markey Cancer Center, University of Kentucky, Lexington, KY 40508, USA

**Keywords:** obesity, NAFLD, insulin resistance, inflammation, UGT1A1, HO-1, bilirubin, urobilin, kinomics, lipidomics

## Abstract

Several population studies have observed lower serum bilirubin levels in patients with non-alcoholic fatty liver disease (NAFLD). Yet, treatments to target this metabolic phenotype have not been explored. Therefore, we designed an N-Acetylgalactosamine (GalNAc) labeled RNAi to target the enzyme that clears bilirubin from the blood, the UGT1A1 glucuronyl enzyme (GNUR). In this study, male C57BL/6J mice were fed a high-fat diet (HFD, 60%) for 30 weeks to induce NAFLD and were treated subcutaneously with GNUR or sham (CTRL) once weekly for six weeks while continuing the HFD. The results show that GNUR treatments significantly raised plasma bilirubin levels and reduced plasma levels of the bilirubin catabolized product, urobilin. We show that GNUR decreased liver fat content and ceramide production via lipidomics and lowered fasting blood glucose and insulin levels. We performed extensive kinase activity analyses using our PamGene PamStation kinome technology and found a reorganization of the kinase pathways and a significant decrease in inflammatory mediators with GNUR versus CTRL treatments. These results demonstrate that GNUR increases plasma bilirubin and reduces plasma urobilin, reducing NAFLD and inflammation and improving overall liver health. These data indicate that UGT1A1 antagonism might serve as a treatment for NAFLD and may improve obesity-associated comorbidities.

## 1. Introduction

Non-alcoholic fatty liver disease (NAFLD) is the most common cause of liver disease in the Western population [1]. The elevating prevalence of NAFLD has followed the rising rates of obesity worldwide, increasing the clinical and economic burden of the disease [1,2,3,4]. NAFLD is rapidly growing in adult populations and becoming a significant health concern in children and teens [5]. NAFLD is also connected to a greater risk of cardiovascular disease. However, the mechanism by which it enhances this risk is not known [2,3,6]. It is becoming accepted that NAFLD is a narrow representation of fatty liver disease whose phenotype is complex, as evidenced by the wide spectrum of disease severity and the wide variability in the patient presentation of the disease. Given the predilection of NAFLD to occur with obesity and other metabolic disorders, such as type II diabetes mellitus, renaming the disease as metabolic dysfunction-associated fatty liver disease (MAFLD) has recently been proposed [7].

It is becoming evident that plasma bilirubin levels are critical for optimal cardiovascular and metabolic health [8]. Higher plasma bilirubin levels have been demonstrated to correlate with protection from cardiovascular disease and diabetes [8,9,10,11,12,13,14,15]. However, the bilirubin catabolized product, urobilin, has been shown to be positively associated with adiposity and insulin resistance in humans [16]. Urobilin is limited by the UGT1A1 enzyme generation of conjugated bilirubin that enters the intestine, where it is deconjugated and formed into the urobilin molecule [15]. Studies in mice show that urobilin is significantly higher in the cecum and liver of obese mice with NAFLD [17]. Others have shown that plasma bilirubin levels are also inversely associated with NAFLD development in children and adults [18,19,20]. Several possible mechanisms by which bilirubin can afford protection against NAFLD include its antioxidant and anti-inflammatory properties [21,22,23,24]. A recent study revealed that bilirubin is a signaling molecule that activates the fat-burning nuclear receptor peroxisome proliferator-activated receptor alpha (PPARα) to stimulate the β-oxidation of fatty acids [25,26,27,28]. The ability of bilirubin to activate PPARα has been proposed as a therapeutic target for treating NAFLD [1,29,30].

Individuals with the Gilbert’s syndrome polymorphism (UGT1A1*28) have reduced expression of the hepatic UGT1A1 enzyme, resulting in mild hyperbilirubinemia [10]. Transgenic mice that are homozygous for the human UGT1A1*28 (HuUGT*28) exhibit moderate hyperbilirubinemia and are protected against the development of dietary-induced hepatic steatosis via decreased serine 73 phosphorylation, an inhibitory site of the PPARα protein [31]. Gilbert’s syndrome patients exhibit decreased fat mass and lowered BMI as compared to healthy controls [32]. Gilbert’s individuals may be protected against the development of obesity and other metabolic disorders by enhanced ATP-dependent AMPK and PPARα activity [33]. Gilbert’s individuals also exhibit decreased inflammatory markers and have enhanced serum antioxidant capacity [34,35]. Because of these data, we hypothesized that targeting UGT1A1 to mimic Gilbert’s syndrome polymorphism might reduce fatty liver disease.

The study aimed to determine if suppressing hepatic UGT1A1 with GNUR could increase plasma bilirubin levels and reduce hepatic lipid accumulation and inflammation in a murine model of dietary-induced NAFLD. We found that GNUR suppressed hepatic UGT1A1 and increased plasma bilirubin, reduced its catabolized product, urobilin [15], and lowered liver fat content and inflammation, improving NAFLD.

## 2. Materials and Methods

### 2.1. Animals

This study’s experimental procedures and protocols conformed to the National Institutes of Health (NIH) Guide for the Care and Use of Laboratory Animals. They were approved by the Institutional Animal Care and Use Committee (IACUC) of the University of Mississippi Medical Center. An electronic laboratory notebook was not used to collect any data in the study. The C57BL/6J male mice were purchased from Jackson Labs (Bar Harbor, ME, USA) and placed on a 60% high-fat diet consisting of 26% protein, 25% carbohydrates, 60% fat in the form of lard and soybean oil, 6% fiber, 6% minerals, and 0.3% vitamins (diet #D12492, Research Diets, Inc., New Brunswick, NJ, USA) for 24 weeks with access to tap water. After this time, mice were randomly assigned to either a subcutaneous treatment consisting of GalNAc-UGT1A1-RNAi (GNUR) (3 mg/kg) or saline containing sham (CTRL) in the exact location between the shoulder blades once a week for 6-weeks while continuing on a high-fat diet. The body composition changes were assessed at 6-week intervals throughout the study, starting at week 18 using magnetic resonance imaging (EchoMRI-900TM, Echo Medical System, Houston, TX, USA). MRI measurements were performed in conscious mice placed in a thin-walled plastic cylinder with a cylindrical plastic insert added to limit the movement of the mice. Mice were briefly submitted to a low-intensity electromagnetic field where fat mass, lean mass, free water, and total water were measured. Food consumption was measured during week 18 of the experimental protocol in mice housed individually. The total amount of food was weighed daily in the morning and averaged for each mouse to obtain a 24-h food consumption measurement. Daily 24-h food consumption measurements were then averaged over one week. The fasting glucose and insulin were measured at baseline and every 2 weeks after initiation of RNAi treatment in mice following an 8-h fast as previously described in [36,37]. The liver composition was measured at the end of the study using magnetic resonance imaging as previously described (EchoMRI-900TM, Echo Medical System, Houston, TX, USA) [36,37]. MRI measurements were performed on whole livers arranged in a thin-walled plastic cylinder. Liver fat and lean mass were obtained and expressed as a percent of total liver weight.

### 2.2. Synthesis of Oligonucleotides

The RNAi targeting *Ugt1a1* was designed and synthesized at Alnylam Pharmaceuticals, as previously described in [38,39]. The siRNA was designed to target mouse *Ugt1A1* mRNA (NM_2016452). It targets position 1673-1693, 5′-GAGAAGUAUUAGUUCAUUAUCUG-3′. Oligonucleotides were synthesized on a MerMade-12 DNA/RNA synthesizer. All oligonucleotides were purified, desalted, and further annealed to form GalNAc-siRNAs as previously described [38].

### 2.3. Liver Triglyceride Measurement

Tissue triglyceride levels were measured using a colorimetric assay kit according to manufacturers’ guidelines (Triglyceride Quantification Colorimetric/Fluorometric Kit, BioVision, Milpitas, CA, USA) as previously described [36,37]. Tissue triglycerides are expressed as mM. Samples from individual mice were run in duplicate and averaged, and the averages were used to obtain group averages.

### 2.4. Plasma Measurements

The plasma total bilirubin levels were measured using a VET AXCEL chemistry analyzer using reagents supplied by the manufacturer (Alpha Wasserman Diagnostic Technologies, West Caldwell, NJ, USA). According to manufacturers’ instructions, the plasma insulin concentrations were determined by ELISA (Linco Insulin ELISA kit, Linco Research Inc., St. Charles, MO, USA). Fasting blood glucose levels were measured using a glucometer. The plasma urobilin was measured by spectrophotometry and performed as described before [16,40,41,42]. In brief, urobilin hydrochloride (Frontier Specialty Chemicals, Logan, UT, USA) was dissolved in DMSO to make the standards (0~125 µM). DMSO/saline was used as the blank for standard/plasma. All blanks, standards, and samples are under the same extraction to form the oxidation products containing urobilin-zinc complexes. Briefly, each tube added 10 µL of standards or plasma samples to 60 µL of 54 mM zinc acetate (in DMSO), followed by 12 µL of 25 mM iodine (dissolved in 120 mM potassium iodine). After mixing for 30 s, 5 µL of 82 mM cysteine HCL was added to each sample, followed by another vortex. Then, centrifuge at 5000× *g* for 3 min. Supernatants were collected into the newly designated tubes, and each pellet underwent repeated extraction. Two supernatants were combined. Hence, 50 µL of 1 M HCL was added into the blanks to eliminate the background fluorescence. The liquid extracted from each sample was added to a 96-well plate, and absorbance was read at 508 nm. Each sample was performed in duplicate. The urobilin concentration of the samples was calculated using the linear regression equation of the standard curve.

### 2.5. Liver Histology

To determine hepatic histological differences, livers were mounted and frozen in Tissue-Tek O.C.T and sectioned at 10 µm as we previously described [43]. Hematoxylin and Eosin (H&E) and periodic acid–Schiff (PAS) staining were performed as previously described [31,36,37,44,45]. Frozen sections were allowed to air-dry and then were fixed in 10% neutral buffered formalin. Sections were briefly rinsed in tap water, followed by 60% isopropanol, and stained for 15 min in the Oil Red O solution. Sections were further rinsed in 60% isopropanol, and nuclei were stained with hematoxylin, followed by aqueous mounting and coverslipping. Slides were imaged at 20× magnification with a color video camera attached to an Olympus VS120 slide scanning microscope (Olympus Corporation, Center Valley, PA, USA). Images were analyzed using the Olympus OlyVIA software. Image J (NIH) was used for quantification. Data are presented as the + SEM for each group.

### 2.6. Lipidomics

Lipids were extracted by acidified organic solvents as described previously [46,47,48]. Lipid class-specific internal standards (Avanti Polar Lipids, Alabaster, AL, USA) were added at the start of the extraction. Dried lipid residues were reconstituted for analysis. The instrument system was a Shimadzu Nexera UHPLC system coupled with an AB Sciex 6500+ Q-Trap linear ion trap/triple quadrupole mass spectrometer. Lipids were separated by HILIC chromatography using a Phenomenex Luna Silica Column with a guard column of the same material and detected in multiple reaction monitoring modes using literature method adaptations. This approach takes advantage of the ability of HILIC chromatography to separate the major classes of glycero and sphingo lipids, allowing for time-scheduled measurements of multiple lipid species within each class. Weakly alkaline solvents (pH 8.0) enhance the detection of anionic lipids in negative-ionization mode. Most lipids were monitored as their precursor molecular ions. In some cases, lipids were monitored as ammoniated adducts. The high sensitivity and speed of the instrument allow for the accurate integration of chromatographic peaks. The method was optimized using a standard mouse-liver lipid extract to identify retention times and exclude lipid species present at low levels and/or not detected consistently. The final optimized method was used to analyze lipids in experimental samples, with data collected for three technical replicates of each sample. Data were analyzed using AB Sciex MultiQuant software (Framingham, MA, USA) for peak finding and integration. The raw peak areas were normalized for recovery of the appropriate internal standards. Lipids species with coefficients of variation of greater than 20% were excluded from the final report.

### 2.7. Lipidomics Data Analysis

LipidSig differential expression analysis tools were used on the web-based platform to identify differentially present lipid species [49]. Before analysis, data were normalized by sample weight for each respective sample. Duplicate species were removed before uploading the normalized values and sample group details into the differential expression online tool. N/A values were classified as undetected but were included in the subsequent analysis. One CTRL sample and two GNUR samples were removed by outlier identification. Lipids with more than 50% missing values across all samples were removed before further analysis. After processing, a total of 1088 lipids remained for analysis across 10 total samples. A *t*-test method was used to identify different species, and a *p*-value < 0.05 was used to determine the significance of differentially present lipids. For global visualization of altered lipid species, hierarchical clustering was performed with a Pearson distance measure and a complete clustering method. Only significantly changed lipid species were analyzed using hierarchical clustering. Differential lipid species are shown using a relative range of signal intensity and were visualized via heatmap. Input data files can be found at the project GitHub repository (https://github.com/evelynb4565/GNUR-Kinome-Analysis-and-Lipidomics, accessed on 6 December 2022).

### 2.8. PamGene PamStation Sample Preparations

Kinase activity was measured using protein tyrosine kinase (PTK) or serine-threonine kinases (STK) PamChip4 porous 3D microarrays and measured using the PamStation12 (PamGene International, ’s-Hertogenbosch, The Netherlands). Substrates contained in each array are included in the project GitHub repository listed above. Mouse livers were pooled and measured in triplicate across three chips simultaneously for PTK and STK, as previously described in [50]. This approach effectively deals with large batch effects across samples. It allows for the characterization of kinase activity only in the context of analytical variance. The pooled samples were lysed using M-PER Mammalian Extraction Buffer (Thermo Fischer Scientific, CAT#78503), Halt Phosphatase Inhibitor (Thermo Fischer Scientific, CAT#78428), and Protease Inhibitor Cocktail (Sigma, CAT#P2714). The samples were homogenized using TissueLyser LT (Qiagen). The protein concentration was measured in triplicate using Pierce BCA Protein assay (Thermo Fischer Scientific, CAT#23225). Samples were diluted to a final protein concentration of 2.5 μg/μL. Each array contained 1 μg of protein per sample for STK chips and 5 μg for PTK chips. In the presence of adenosine triphosphate (ATP), kinase phosphorylation activity is quantified using fluorescently labeled antibodies to detect differential phosphorylation of 196 (PTK) or 144 (STK) reporter peptides between experimental and control conditions, as previously described [50]. Evolve (PamGene) software uses a charge-coupled device (CCD) camera and light-emitting diode (LED) imaging system to record relative phosphorylation levels of each unique consensus phosphopeptide sequence every 5 min for 60 min as measured by peptide signal intensities recorded across 10, 20, 50, and 100 millisecond exposure times. Raw imaging data were exported for further data analysis and kinase mapping.

### 2.9. PamGene PamStation Kinome Data Analysis

The images taken during the run were analyzed using BioNavigator (PamGene). Signal ratios are used to calculate fold change (FC) for each phosphopeptide sequence averaged across three replicates. Minimum threshold values were selected using cutoffs cited in previous literature [50,51,52,53]. These thresholds require differential phosphopeptide signals greater than or equal to 30% (FC ≥ 1.30 or FC ≤ 0.70) for differential phosphorylation to be considered. Linear regression slopes provide phosphorylation intensity signals used in differential analyses (e.g., experimental vs. control). Undetectable and/or nonlinear (R^2^ < 0.80) phosphopeptides are excluded from subsequent analyses. We performed upstream kinase identification using Kinome Random Sampling Analyzer (K.R.S.A.) [54] and Upstream Kinase Analysis (U.K.A.) [55], as previously described in [56]. The kinase scores from the K.R.S.A. and U.K.A. are included in the project GitHub repository. MEOW (measurements extensively of winners) plots were used to interrogate individual kinase activities on substrates considering the confidence of the experimental versus the control groups using the equation: [Log2 Fold Change (FC) of kinase substrates * Δconfidence (experimental hits/mean hits of 2000 random sampling iterations)]. The kinome phyla tree was made using CORAL [57]. R Markdown files for the K.R.S.A. R package are available on the GitHub repository at the following (https://github.com/evelynb4565/GNUR-Kinome-Analysis-and-Lipidomics, accessed on 6 December 2022).

### 2.10. Quantitative Real-Time PCR Analysis

Total RNA was harvested from the livers using a Qiagen Tissue Lyser LT (Qiagen) and then extracted using the RNeasy kit (Qiagen). We quantitated the total RNA on a NanoDrop 2000 spectrophotometer (Thermo Fisher Scientific, Wilmington, DE, USA), and cDNA was synthesized using High-Capacity cDNA Reverse Transcription Kit (Applied Biosystems). PCR amplification of the cDNA was performed by quantitative real-time PCR for gene-specific primers as previously described using TrueAmp SYBR Green qPCR SuperMix (Alkali Scientific). The thermocycling protocol consisted of 5 min at 95 °C, 40 cycles of 15 s at 95 °C, and 30 s at 60 °C, finished with a melting curve ranging from 60 to 95 °C to allow the distinction of specific products. Normalization was performed in separate reactions with primers to 36B4.

### 2.11. Western Blotting

Mouse liver tissues were flash-frozen in liquid nitrogen during harvesting and stored at −80 °C. For gel electrophoresis, 30 mg of cut tissue was resuspended in 3 volumes of M-PER Mammalian Protein Extraction Reagent (ThermoFisher Scientific, Cat no: 78501) plus 10% protease inhibitor cocktail (Sigma P2714-1BTL) and Halt phosphatase inhibitor cocktail (Fisher PI78420), and then incubated on ice for 30 min. The samples were lysed with a Qiagen Tissue Lyser LT (Qiagen) and centrifuged at 100,000× *g* at 4 °C. Protein samples (30 μg) were resolved by SDS polyacrylamide gel electrophoresis and electrophoretically transferred to Immobilon-FL membranes. Membranes were blocked at room temperature for 1 h in the LI-COR Intercept Blocking Buffer (LI-COR Biosciences, Cat no: 927-60001). Subsequently, the membranes were incubated overnight at 4 °C with antibody against UGT1A1 (Invitrogen, Cat no PA5-76862: 1:1000 dilution in LI-COR Intercept Antibody Diluent T20-TBS) and heat shock protein 90 (HSP90) (Santa Cruz Biotechnology, Cat no: sc-13119; 1:10,000 dilution in LI-COR Intercept Antibody Diluent T20-TBS). After three washes in TBS + 0.1% Tween 20, membranes were incubated with an infrared anti-rabbit (IRDye 800, green) or anti-mouse (IRDye 680, red) secondary antibody labeled with IRDye infrared dye (LI-COR Biosciences) (1:10,000 dilution in LI-COR Intercept antibody diluent T20-TBS) for 2 h at 4 °C. Immunoreactivity was visualized and quantified by infrared scanning in the Odyssey system (LI-COR Biosciences, Lincoln, NE, USA).

### 2.12. Statistics

All data are presented as mean + S.E.M. Differences between treatment groups were determined using the student *t*-test or one-way analysis of variance with a post hoc test (Dunnett’s). A *p* < 0.05 was considered significant. Statistical analysis was performed with GraphPad Prism 9 software (GraphPad Software, Inc., San Diego, CA, USA). All kinase data produced from the PamStation was first processed and analyzed by Bionavigator software, which provides data for individual kinases. Kinase data were also analyzed by the KRSA package (kinase random sampling analysis) (https://github.com/CogDisResLab/KRSA) in R (version 4.1.2), which is suited for both individual and kinase families. Results from both pipelines were used and presented in the present manuscript.

## 3. Results

### 3.1. Suppression of Hepatic UGT1A1 Increases Bilirubin Levels and Lean Mass and Lowers Blood Glucose and Insulin Levels

**Figure 1:**
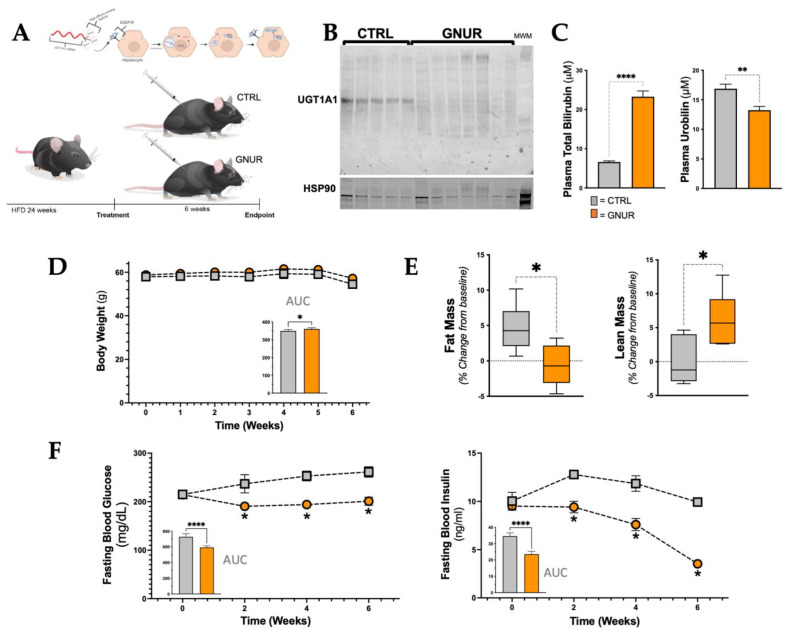
Suppression of hepatic UGT1A1 increases plasma bilirubin levels and lean mass and lowers plasma levels of urobilin, glucose, and insulin. (**A**) Experimental design. Male C57/bl6 mice were fed a high-fat diet (HFD) for 24 weeks and then treated with CTRL or GNUR subcutaneously for 6 additional weeks while maintaining the HFD. (**B**) Western blotting of UGT1A1 and heat shock protein 90 (HSP90) in CTRL and GNUR treated mice. (**C**) Plasma levels for total bilirubin and the bilirubin catabolized product urobilin [n = 4 for each group for bilirubin; n = 6 for each group for urobilin, **, *p* < 0.01; ****, *p* < 0.0001]. (**D**) Body weights were measured weekly, and the area under the curve (AUC) was calculated [n = 6–7 for each group; *, *p* < 0.05]. (**E**) Measurement of fat and lean masses via echo MRI [n = 6 for each group; *, *p* < 0.05]. (**F**) fasting blood glucose and insulin levels and AUC was calculated and graphed [n = 6–7 for each group; *, *p* < 0.05].

In the present study, we tested the ability of GNUR to increase plasma bilirubin as a potential therapy for NAFLD. The GNUR and CTRL were administered once weekly via subcutaneous injection for six weeks in mice with established dietary-induced obesity (Figure 1A). At the end of this time, hepatic UGT1A1 protein expression was measured by Western blotting using heat shock protein 90 (HSP90) as the control (Figure 1B). The weekly GNUR treatment resulted in significantly (*p* < 0.001) higher plasma total bilirubin levels (Figure 1C). The bilirubin catabolized product, urobilin, was significantly (*p* < 0.001) lower in the plasma with GNUR treatment compared to CTRL (Figure 1C). To determine whether GNUR affects metabolic parameters, we first measured body weight, fat and lean mass, and fasting blood glucose and insulin levels. The body weights were significantly increased with the GNUR treatment compared to CTRL (Figure 1D). While the weight was increased, echo MRI analysis showed that fat mass was significantly (*p* < 0.05) reduced with GNUR treatments, and lean mass was significantly higher (Figure 1E). The fasting blood glucose levels were similar in both groups prior to the start of treatment (215 ± 4 vs. 214 ± 4 mg/dL, CTRL vs. GNUR). After the treatments, the GNUR treatment resulted in significantly (*p* < 0.05) decreased fasting blood glucose, which was sustained until the end of the experimental protocol at six weeks of treatment (Figure 1F). Likewise, fasting blood insulin levels were similar between both groups before the initiation of GNUR treatment (10 ± 1 vs. 9.5 ± 0.5 ng/mL, CTRL vs. GNUR). The GNUR treatment significantly decreased (*p* < 0.05) fasting blood insulin levels over the entire treatment period (Figure 1F).

### 3.2. Suppression of UGT1A1 Decreases Hepatic Steatosis, Inflammation, and Triglyceride Levels

**Figure 2:**
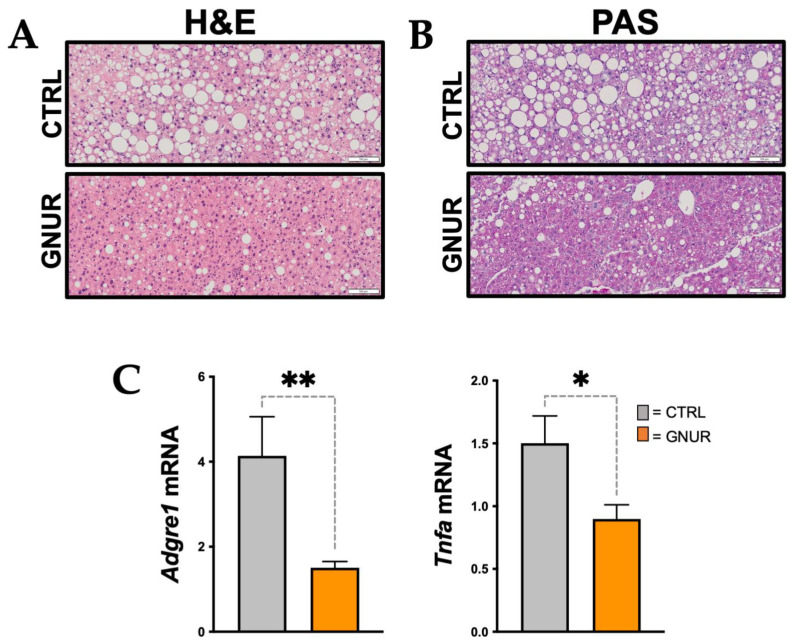
Liver histology and expression of inflammatory markers in GNUR- and CTRL-treated obese mice. (**A**) Hematoxylin and Eosin (H&E) and (**B**) Periodic acid-Schiff (PAS) staining of the livers from GNUR- and CTRL-treated obese mice [scale bar = 100 μm]. (**C**) Inflammatory markers *Adgre1* (F480) and *Tnfa* mRNA expression in the livers from GNUR- and CTRL-treated obese mice [n = 6–7 for each group; *, *p* < 0.05; **, *p* < 0.01].

To determine how GNUR and CTRL treatments affected the liver, we next performed histology and measured hepatic steatosis and inflammatory markers at the end of the experimental protocol. The histological analysis using Hematoxylin and Eosin (H&E) and periodic acid–Schiff (PAS) staining of the livers shows the presence of lipid droplets (white spaces) in CTRL and that these are reduced with GNUR treatment (Figure 2A,B). The PAS staining had more purple coloring in the GNUR compared to CTRL-treated animals (Figure 2B), which suggests that the glycogen content in the obese mice with GNUR treatment might be higher compared to the CTRL treated. Liver glycogen content is regulated by insulin sensitivity [36], and the GNUR-treated animals did exhibit lower blood glucose and insulin levels, indicating that they might be more insulin sensitive. In Figure 2C, we show that inflammatory makers Adgre1 (also known as F480) and Tnfa mRNA levels were significantly reduced with the GNUR compared to the CTRL-treated group. We have previously shown that increasing plasma bilirubin reduces hepatic inflammation [31,43].

**Figure 3:**
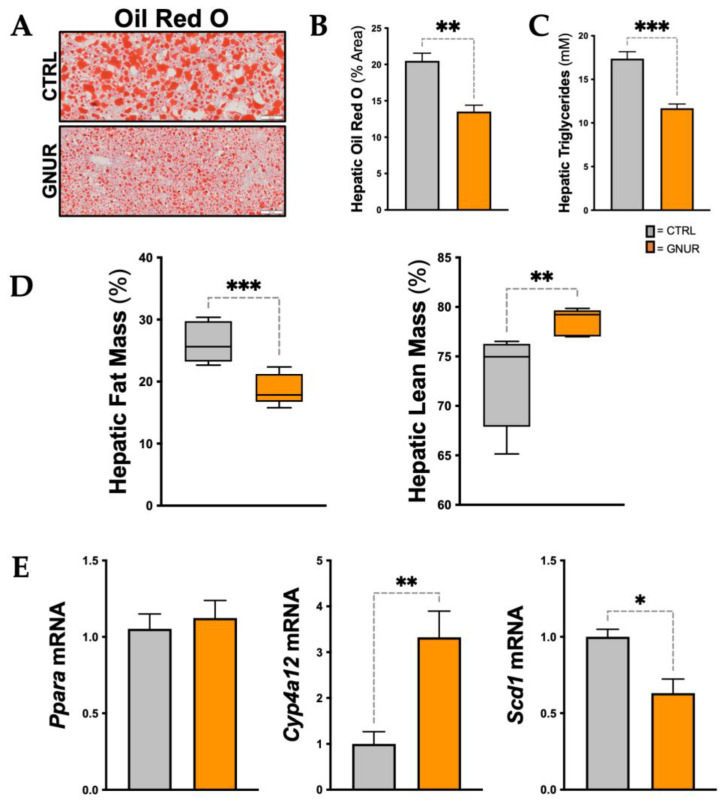
Hepatic lipid accumulation and PPARα signaling in the liver. (**A**) Oil Red O staining of the livers from GNUR- and CTRL-treated obese mice [scale bar = 100 μm], and (**B**) Oil Red O staining quantification [n = 4 for each group; **, *p* < 0.01]. (**C**) biochemical measurement of triglycerides in both groups [n = 4 for each group; ***, *p* < 0.001]. (**D**) Echo MRI measurements of hepatic fat and lean masses for GNUR- and CTRL-treated obese mice [n = 6–7 for each group; **, *p* < 0.01; ***, *p* < 0.001]. (**E**) Real-time PCR measurement of Ppara, Cyp4a12, and Scd1 mRNA expression in livers from GNUR- and CTRL-treated obese mice [n = 5–7 for each group; *, *p* < 0.05; **, *p* < 0.01].

Similar to the H&E and PAS staining above, Oil Red O staining of the liver sections also demonstrated significantly (*p* < 0.01) decreased hepatic steatosis in GNUR-treated mice compared to CTRL (20.5 ± 2 vs. 13.5 ± 1.5% area, CTRL vs. GNUR) (Figure 3A,B), and as measured biochemically (17.4 ± 0.5 vs. 11.7 ± 0.3 mM, CTRL vs. GNUR) (Figure 3C). The EchoMRI analysis revealed that GNUR treatment resulted in significantly (*p* < 0.001) decreased hepatic fat mass (26.2 ± 1.5 vs. 18.8 ± 1%, CTRL vs. GNUR) and significantly (*p* < 0.01) increased hepatic lean mass (72.8 ± 2 vs. 78.5 ± 0.5%, CTRL vs. GNUR) (Figure 3D). Previous reports have shown that increasing plasma bilirubin in obese mice activated PPARα signaling mechanisms [26,28,29,31,44]. Therefore, we measured hepatic PPARα (Ppara) mRNA levels, and there were no significant differences observed between GNUR and CTRL groups (Figure 3E). However, the level of PPARα does not depict its transcriptional activity. Therefore, we measured known PPARα regulated genes Cyp4a12 and Scd1 [31,36]. The expression of Cyp4a12 was significantly (*p* < 0.01) increased with GNUR compared to CTRL treatments, and Scd1 decreased (*p* < 0.05). The actions of Scd1 are supposed to increase hepatic de novo lipogenesis, and suppression by GNUR treatment significantly reduced hepatic fat, as observed in the Oil Red O and H&E liver staining.

### 3.3. Lipidomics Reveals Decreased Ceramide Lipid Accumulation in the Liver of GNUR-Treated Mice

**Figure 4:**
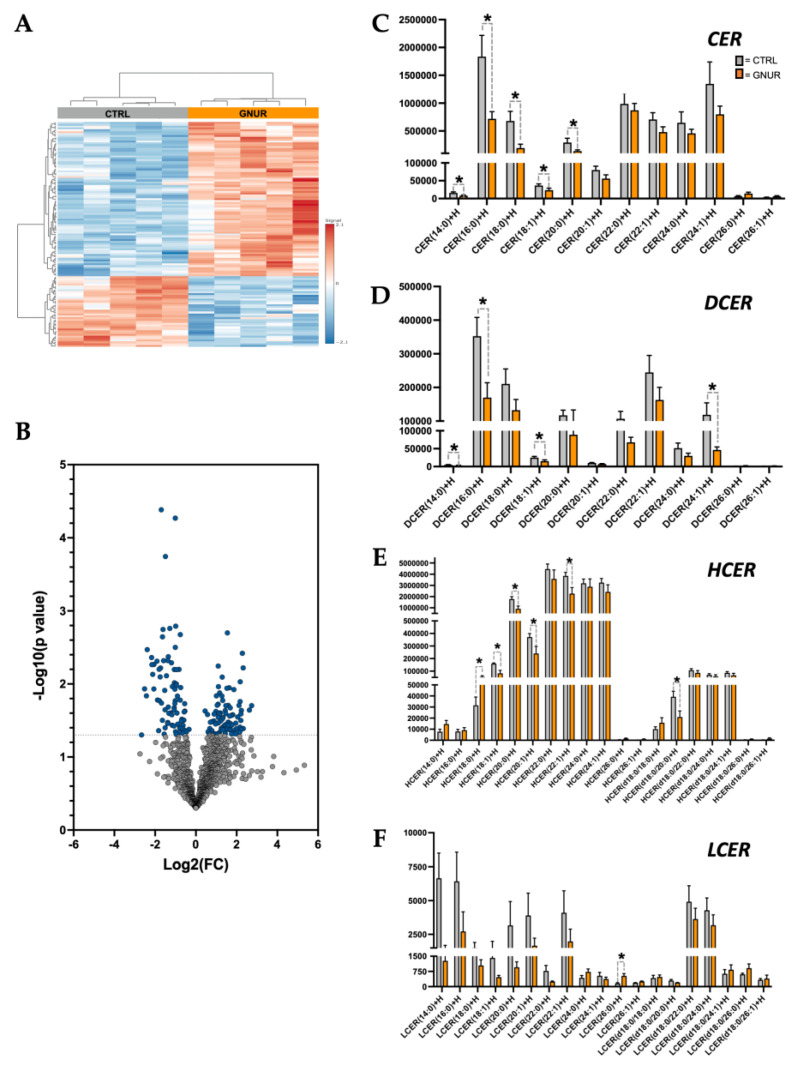
Lipidomics analysis of lipid species from the livers of GNUR- and CTRL-treated obese mice. (**A**) The heatmap of lipid species significantly changed in GNUR- and CTRL-treated obese mice. (**B**) Volcano plot of lipid species from lipidomics analysis of livers from GNUR- and CTRL-treated obese mice. (**C**–**F**) Sub-lipid species levels from four ceramide classes: ceramide (CER), dihydroceramide (DCER), hexosylceramide (HCER), and lactosylceramide (LCER) from GNUR- and CTRL-treated obese mice. [minimal n = 5 per group; *, *p* < 0.05].

The results above indicate that GNUR treatment improves NAFLD, particularly in the decrease in percent hepatic fat mass and lipid accumulation. Therefore, we wanted to explore the types of lipids present in the GNUR- and CTRL-treated groups and how the lipid species differed with reduced NAFLD. We, therefore, performed lipidomics using liquid-chromatography-mass spectrometry (LC-MS) as we have previously described in [58] to analyze the lipid species from hepatic tissues from both GNUR- and CTRL-treated groups. We detected a total of 1143 lipid species, among which 163 lipids were significantly changed in the GNUR-treated compared to CTRL (*p*-value < 0.05). Heatmap analysis of the lipid species from the livers of the GNUR- and CTRL-treated mice showed clear, distinct differences in lipid species (Figure 4A), which was also supported by the volcano plot (Figure 4B). The volcano plot analysis revealed that the ceramide (CER) species was generally decreased overall. Therefore, we analyzed the individual species of this lipid class to determine differences in each subgroup. We found significant changes in four ceramide classes: CER, DCER, HCER, and LCER (Figure 4C–F). Interestingly, we found that the shorter-chain lipids were significantly decreased, and only a limited number of longer-chain lipids were significantly changed. These were specifically the CER 14:0, CER 16:0, CER 18:0, and CER 20:0 lipid species that were significantly lower in GNUR-treated compared to CTRL (*p*-value < 0.05). Whereas the longer chain species (i.e., CER 22:0 and higher) were either not significantly reduced in the GNUR-treated or unchanged compared to CTRL-treated.

### 3.4. Kinome Signaling Analysis

**Figure 5:**
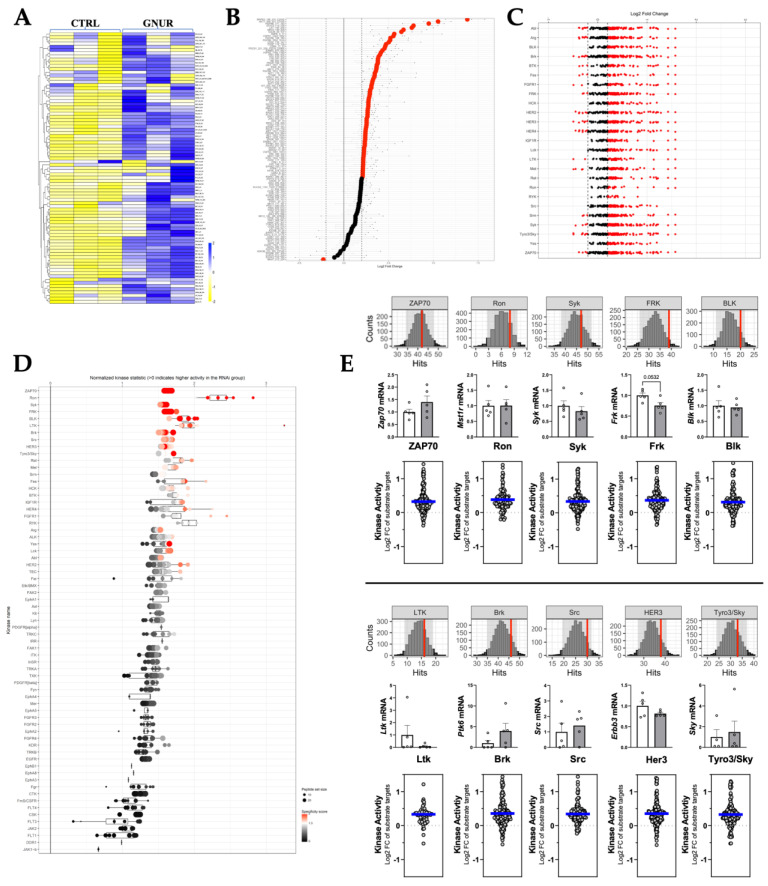
Phospho-tyrosine kinase (PTK) PamGene kinome activity analysis. (**A**) Heatmap analysis of differential PTK substrate phosphorylation. (**B**) Waterfall plots are shown by log fold change of substrate phosphorylation. (**C**) Differentially phosphorylated substrates mapped back to responsible kinases (upstream analysis) by reverse KRSA analysis. (**D**) Waterfall plot of individual kinases. (**E**) Peacock plots (confidence measure indicated by the red line), mRNA measurements, and MEOW plots for the top kinases.

Next, as we have previously described for our kinome analysis of humans with liver cirrhosis [50], we wanted to determine the kinase signaling responsible for the improvement of NAFLD in the GNUR-treated compared to the CTRL-treated group. Therefore, we measure the kinase activity of phospho-tyrosine kinases (PTKs) and serine-threonine kinases (STKs) using PamGene PamStation technology, as we described in [50]. Our PTK analysis revealed increased substrate phosphorylation activity in the GNUR-treated compared to the CTRL-treated group, as shown in the heatmap and waterfall plot analysis (Figure 5A,B). Analysis of the PTK substrate phosphorylation indicated that the activity of many PTK kinases was higher (Figure 5C). Upstream pathway analysis shown in the waterfall plot in Figure 5D indicated that several PTK pathways were hyperactive, as indicated by the red-pink coloring (Figure 5D). We further scrutinized the top targets changed and quantitated peacock plots (histograms with the red line as a measure of confidence) and found that all had higher confidence of being significantly changed, as indicated by the red line (Figure 5E, top figure). Next, we measured mRNA expression by real-time PCR and found no significant changes in the expression of any of the PTK kinases (Figure 5E, middle figure). Lastly, we measured the MEOW plot analysis for kinase activity. We observed that all of the kinases were indeed significantly changed, as indicated by the kinase activity (blue line) in the graph (Figure 5E, bottom figure).

**Figure 6:**
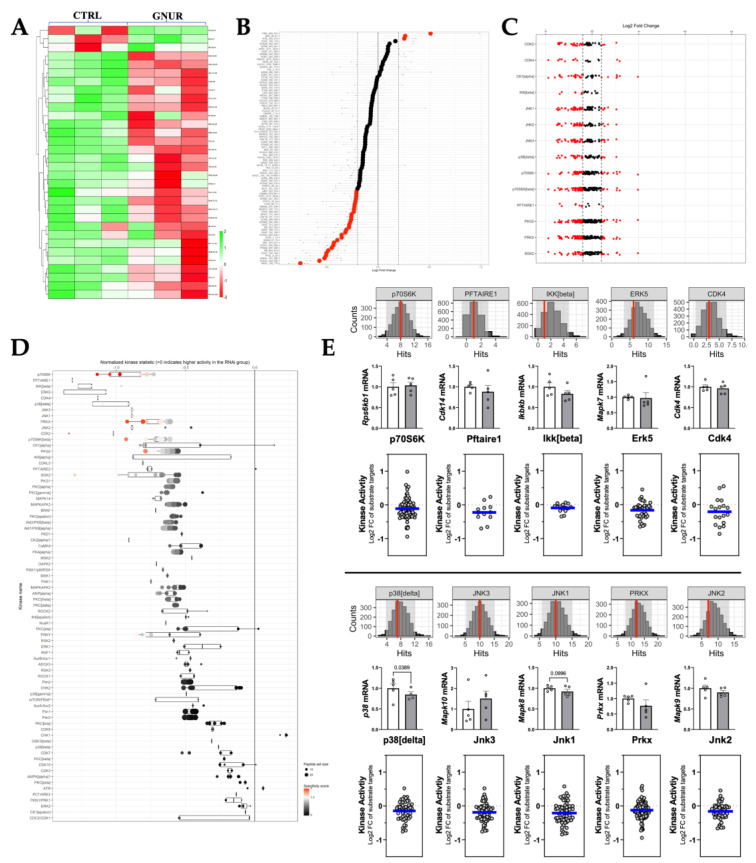
Serine/threonine kinase (STK) PamGene kinome activity analysis. (**A**) Heatmap analysis of differential STK substrate phosphorylation. (**B**) Waterfall plots are shown by log fold change of substrate phosphorylation. (**C**) Differentially phosphorylated substrates mapped back to responsible kinases (upstream analysis) by reverse KRSA analysis. (**D**) Waterfall plot of individual kinases. (**E**) Peacock plots (confidence measure indicated by red line), mRNA measurements, and MEOW plots for the top kinases.

**Figure 7:**
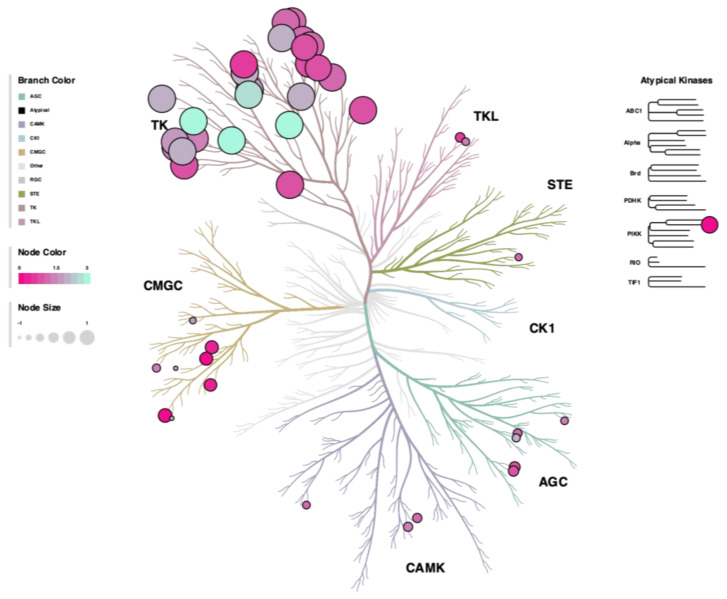
Kinase phylogeny tree of livers from GNUR-treated compared to CTRL-treated obese mice. Both STK and PTK results are included, where node size refers to the calculated mean final score and the node color refers to the median kinase statistic. Both measures are calculated by Bionavigator software (https://pamgene.com/technology/). The phylogeny tree was created using the CORAL software (https://github.com/dphansti/CORAL).

The STK analysis showed a general hypophosphorylation across assayed substrates shown in the heatmap and waterfall plot analysis (Figure 6A,B), as also shown in the reverse KRSA plot (Figure 6C) and for individual kinase activity in the upstream STK waterfall plot (Figure 6D). We further quantitated peacock plots of the top targets changed and observed that all had moderate confidence of being significantly changed, as indicated by the red line (Figure 6E, top figure). Next, we measured mRNA expression by Real-time PCR. We found that only p38 (delta) was significantly lower, and no other changes in the expression of any of the STK kinases were observed (Figure 6E, middle figure). Lastly, we measured the MEOW plot analysis for kinase activity. The kinase activity, as indicated by the blue line, was only moderately reduced in the graph (Figure 6E, bottom figure). A phyla tree in Figure 7 represents the overall kinase changes.

## 4. Discussion

The high rates of obesity have spawned an increase in NAFLD [1,2,3,4]. The epidemic rise in NAFLD rates mandates the development of novel therapies that can reverse hepatic steatosis and prevent the acceleration of the condition to non-alcoholic steatohepatitis (NASH) or, ultimately, liver cirrhosis or cancer. The results of numerous clinical studies demonstrate that plasma levels of bilirubin inversely correlate with the development of NAFLD and NASH, indicating that increasing plasma bilirubin might be a logical target for the treatment of NAFLD [19,59,60,61]. A recent study in humans showed that urobilin, which is formed from the catabolism of conjugated bilirubin by the microbiome in the gut [15], was significantly higher in obese men and women and was positively associated with insulin resistance [16]. Our studies here indicate that suppressing UGT1A1 in the liver lowers plasma urobilin and increases plasma bilirubin levels, improving overall liver health. Several studies have proposed the activation of the heme oxygenase pathway (HO-1) as a means of providing metabolic protection [62,63]. We show here that another approach to regulating plasma bilirubin levels is to target hepatic UGT1A1, which is the enzyme responsible for the conjugation of bilirubin in the liver for its elimination in the bile [8,64].

In the present study, we chose to target hepatic UGT1A1 via the administration of GNUR that suppressed expression. The GNUR treatments were highly effective in reducing hepatic UGT1A1, decreasing the levels by greater than 90% compared to CTRL-treated animals. This resulted in a significant increase in plasma bilirubin levels by about 10-fold and a reduction of the bilirubin catabolized product, urobilin, that is absorbed from the gut via the hepatic portal vein [15]. The GNUR-treated animals did have significantly less hepatic fat content compared to the CTRL-treated animals, as measured by echo MRI, biochemically, and Oil Red O and H&E staining of the livers. The lipidomics analysis showed that GNUR treatments significantly reduced ceramide production via the hepatic de novo lipogenesis pathway.

Studies in mice with the human Gilbert’s syndrome polymorphism showed that these mice were resistant to dietary-induced obesity hepatic steatosis with only a mild elevation (two-fold) in plasma bilirubin levels [31]. The increase in plasma bilirubin levels observed in the present study (~10-fold) reflects the high degree of inhibition of hepatic UGT1A1 by GNUR, which decreased hepatic steatosis in our model of dietary-induced obesity. We have previously found that hyperbilirubinemia due to Gilbert’s polymorphism also attenuated dietary obesity-induced hepatic steatosis [31]. Similar to our other findings with mild hyperbilirubinemia, GNUR-treated obese mice had activated PPARα pathways that reduced hepatic lipid content and increased liver glycogen. GNUR treatment in this model offers an advantage as the effects of hyperbilirubinemia can be evaluated in a model of established NAFLD, which is more clinically relevant than the mouse Gilbert’s model, which is a model of congenital hyperbilirubinemia.

The GNUR treatments also significantly lowered fasting blood glucose and insulin levels in our model of dietary-induced obesity. The effect on fasting blood glucose and insulin levels was similar to that observed in previous studies in both dietary-induced obese mice as well as leptin receptor-deficient *db/db* mice treated with bilirubin [23,65]. Studies on the enzyme that generates bilirubin, biliverdin reductase A (BVRA), have shown that the loss of the localized production of bilirubin in the liver and adipose tissues causes metabolic stress and fat accumulation and oxidative stress [36,66,67,68,69,70]. These preclinical analyses have been validated in human investigations, which show that BVRA is lower in patients with metabolic dysfunction [71,72]. Studies have also shown that loss of BVRA causes insulin resistance in the brain that may be linked to Alzheimer’s disease [73,74,75,76]. Exercise improves metabolic dysfunction, increases BVRA, and lowers UGT1A1 expression in the livers of high-capacity running rats compared to low-capacity runners [44]. Some studies have proposed that bilirubin might benefit exercise and performance [24,77]. This concept also agrees with the many human population studies in which plasma levels of bilirubin are correlated with protection against the development of diabetes in a wide range of patient populations [78,79,80,81]. There are several potential mechanisms by which bilirubin improves insulin sensitivity, including alterations in skeletal muscle PKB/Akt signaling, suppression of macrophage infiltration, and inflammatory cytokine release from adipose, as well as suppression of hepatic endoplasmic reticulum (ER) stress [23,65]. With our advanced kinome analysis, we were able to highlight several kinases that may help to explain some of the clear metabolic improvements induced by the GNUR treatment.

Our PTK analysis revealed hyperactivation of the recepteur d’origine nantais (RON) kinase. The RON kinase has only one known ligand, macrophage stimulating protein (MSP) [82], which is predominately expressed in the liver. Once MSP is bound to RON, it acts by inhibiting nitric oxide synthesis (NOS), reducing inflammatory responses [82]. MSP-RON signaling is considered to be a marker of liver inflammation [82], and its hyperactivity was detected in our analysis, suggesting it plays an important role in mediating the reversal of NAFLD-associated pathologies. Some of the most changed STK kinases had a high specificity score, such as P38, JNK, and p70s6k kinases, which are known to be involved in mediating a number of inflammatory-associated pathways, and they were found to be lower with the GNUR compared to the CTRL treatments. These hypoactive kinases are linked to the significantly decreased mRNA expression of inflammatory markers *Adgre1* and *Tnfa*.

Bilirubin can also improve insulin sensitivity through the PPARα pathway through increased plasma levels of fibroblast growth factor 21 (FGF21) [28,83]. It is possible that bilirubin can also improve insulin sensitivity by direct action on pancreatic beta cells [84,85]. Bilirubin likely acts through several of these pathways to improve insulin sensitivity [8,86]. It is important to note the relatively short treatment period that was used in the present study and the impressive effects that were observed on fasting blood glucose and insulin as well as hepatic steatosis. It is possible that greater improvements in these phenotypes could be achieved with longer treatment.

One limitation of the overall strategy of targeting hepatic UGT1A1 to increase plasma bilirubin levels for the treatment of NAFLD is the wide range of substrates of UGT1A1, which was also indicated by the kinome analysis. The glucuronyl UGT1A1 enzyme is responsible for the conjugation of bilirubin in the liver but also plays an important role in the metabolism of drugs and other compounds by the liver [64,87]. Given the fact that most patients with NAFLD also have other chronic diseases, such as hypertension and diabetes, it is likely that these patients would be taking medications for these conditions as well. Therefore, specific care would need to be taken to achieve a level of UGT1A1 inhibition, which raises plasma bilirubin but does not deplete hepatic UGT1A1 completely so that it can metabolize any other medications taken by this patient population. These types of issues with inhibitors of hepatic UGT1A1 need to be fully considered before the translation of this approach to human patient populations.

While the observed increase in plasma total bilirubin levels in the present study was high, it is still far below levels that would result in clinical jaundice. Traditionally, plasma bilirubin levels in jaundice exceed 3 mg/dL (>51 μM) [88], and clinical jaundice does not manifest until over 100 μM [30]. We have previously demonstrated that even milder increases in plasma bilirubin (50%-2 fold) can protect against angiotensin II-induced hypertension and increased renal vascular resistance [89,90]. In this study with GNUR, we did not observe any adverse phenotypes in our mice, such as excessive itching, as bilirubin has recently been reported to activate the Mas-related G-protein Coupled Receptor (MRGPR) to mediate cholestatic itch at very high levels (>150 μM) [91].

In conclusion, our results demonstrate that suppressing hepatic UGT1A1 with GNUR increases plasma bilirubin levels, lowers the bilirubin catabolized product urobilin that is absorbed from the gut, reverses hepatic steatosis, and improves fasting hyperglycemia and hyperinsulinemia in a mouse model of established dietary-induced obesity and NAFLD. This approach could be tailored to patients exhibiting the metabolic syndrome to lower hepatic steatosis and reverse insulin resistance. Future studies to determine factors that reduce urobilin and increase bilirubin in the plasma may be beneficial to those with metabolic, inflammatory, and cardiovascular diseases.

## Data Availability

All data and supplemental information regarding this manuscript is included on GitHub or can be provided upon request.
